# Predicting outcomes in chronic kidney disease: needs and preferences of patients and nephrologists

**DOI:** 10.1186/s12882-023-03115-3

**Published:** 2023-03-22

**Authors:** Dorinde E. M. van der Horst, Noel Engels, Jos Hendrikx, Marinus A. van den Dorpel, Arwen H. Pieterse, Anne M. Stiggelbout, Cornelia F. van Uden–Kraan, Willem jan W. Bos

**Affiliations:** 1 Santeon, Utrecht, The Netherlands; 2 Department of Internal Medicine, Leiden University Medical Centre, Leiden, the Netherlands; 3 Department of Internal Medicine, St. Antonius Hospital, Nieuwegein, the Netherlands; 4 Department of Internal Medicine, Maasstad Hospital, Rotterdam, the Netherlands; 5 Department of Biomedical Data Sciences, Leiden University Medical Centre, Leiden, The Netherlands; 6 Erasmus School of Health Policy and Management, Erasmus University Rotterdam, Rotterdam, The Netherlands

**Keywords:** Prediction models, Chronic kidney disease, Decision making, Patient outcomes

## Abstract

**Introduction:**

Guidelines on chronic kidney disease (CKD) recommend that nephrologists use clinical prediction models (CPMs). However, the actual use of CPMs seems limited in clinical practice. We conducted a national survey study to evaluate: 1) to what extent CPMs are used in Dutch CKD practice, 2) patients’ and nephrologists’ needs and preferences regarding predictions in CKD, and 3) determinants that may affect the adoption of CPMs in clinical practice.

**Methods:**

We conducted semi-structured interviews with CKD patients to inform the development of two online surveys; one for CKD patients and one for nephrologists. Survey participants were recruited through the Dutch Kidney Patient Association and the Dutch Federation of Nephrology.

**Results:**

A total of 126 patients and 50 nephrologists responded to the surveys. Most patients (89%) reported they had discussed predictions with their nephrologists. They most frequently discussed predictions regarded CKD progression: when they were expected to need kidney replacement therapy (KRT) (*n* = 81), and how rapidly their kidney function was expected to decline (*n* = 68). Half of the nephrologists (52%) reported to use CPMs in clinical practice, in particular CPMs predicting the risk of cardiovascular disease. Almost all nephrologists (98%) reported discussing expected CKD trajectories with their patients; even those that did not use CPMs (42%). The majority of patients (61%) and nephrologists (84%) chose a CPM predicting when patients would need KRT in the future as the most important prediction. However, a small portion of patients indicated they did not want to be informed on predictions regarding CKD progression at all (10–15%). Nephrologists not using CPMs (42%) reported they did not know CPMs they could use or felt that they had insufficient knowledge regarding CPMs. According to the nephrologists, the most important determinants for the adoption of CPMs in clinical practice were: 1) understandability for patients, 2) integration as standard of care, 3) the clinical relevance.

**Conclusion:**

Even though the majority of patients in Dutch CKD practice reported discussing predictions with their nephrologists, CPMs are infrequently used for this purpose. Both patients and nephrologists considered a CPM predicting CKD progression most important to discuss. Increasing awareness about existing CPMs that predict CKD progression may result in increased adoption in clinical practice. When using CPMs regarding CKD progression, nephrologists should ask whether patients want to hear predictions beforehand, since individual patients’ preferences vary.

**Supplementary Information:**

The online version contains supplementary material available at 10.1186/s12882-023-03115-3.

## Introduction

The course of chronic kidney disease (CKD) and the risk of progression to end-stage kidney disease (ESKD) vary among patients [1–3]. Guidelines recommend that nephrologists use clinical prediction models (CPMs) to help identify patients at increased risk of CKD progression and adjust their treatment to help limit further kidney function decline [2, 3]. In addition, multiple studies showed that patients are interested in prognostic information, and that they value this information for behavioural change and treatment planning [4–6]. CPMs can also be used to help establish the optimal timing of starting education on kidney replacement therapy (KRT) when patients do progress to the more advanced stages of CKD. Timely education and decisional support allow for effective decision-making, and may prevent delays in the decision-making process which are associated with increased patient morbidity, mortality and health care costs [7].

Numerous CPMs have been developed for CKD practice over the years. These include models that predict the risk of progression to ESKD [8–16] or adverse outcomes of different KRT modalities, such as: 1) mortality after dialysis initiation [17–34], and 2) rejection after kidney transplantation [35, 36]. Some of these models, such as the Kidney Failure Risk Equation (KFRE), have been extensively validated and offer good predictive performance [9–11, 37–41]. Even though well-validated models are readily available and guidelines recommend that nephrologists use CPMs, the actual use of CPMs in CKD practice seems limited [6, 42–44]. This may be related to the CPMs themselves (e.g., limitations in predictive performance or user friendliness), and/or to the intended users (e.g., doubts about the reliability and generalizability of CPMs) [43, 44]. CPMs are also often developed without the input of end-users (i.e., patients and nephrologists), and as a consequence, lack clinical relevance [42, 43]. In addition, patients and nephrologists often prioritize different (treatment) outcomes [45, 46] and may have different needs and preferences regarding the use and purpose of CPMs in CKD practice.

Therefore, the aim of this study was to: 1) evaluate to what extent CPMs are currently used in the Dutch CKD practice, 2) identify patients’ and nephrologists’ needs and preferences regarding predictions in CKD, and 3) explore determinants that may affect the adoption of CPMs in CKD practice. Our results can be used to guide implementation of CPMs and inform future development of CPMs.

## Material and methods

### Study design

A national survey study among CKD patients and nephrologists in The Netherlands was conducted. First, patients’ attitudes towards different CPMs predicting the course of CKD were explored in semi-structured interviews. Next, two online surveys were developed and distributed: one for patients and one for nephrologists.

### Semi-structured interviews

Patients with CKD were interviewed to explore their attitudes towards the use of CPMs in CKD practice. These interviews were held in the context of a larger study on the development of a CKD dashboard [47]. During these interviews, two different predictions were introduced: 1) the prediction from the KFRE: a 2- and 5-year risk of progression to kidney failure for stages 3 to 5 CKD patients (in %), and 2) a prediction about the time until kidney failure (in years). Mock-ups were used to present these predictions in a similar lay-out to have patients focus on the meaning of the predictions rather than on how these were presented (Additional file 1). Patients were asked to ‘think-out-loud’ and give their first impressions on the presented predictions. Patients were subsequently asked whether they would want to be provided with these predictions in (including reasons why), and how they would prefer to receive this information.

### Online surveys

Two surveys were developed: one for CKD patients and one for nephrologists. Each survey started with an introductory text and an explanation of the definition of a CPM. This explanation was supplemented with an infographic to facilitate understanding (Additional file 2). Both surveys consisted of questions assessing: 1) the current use of CPMs in Dutch CKD practice, 2) preferences for predictions in CKD, 3) preferences for predictions about CKD progression (to ESKD), and 4) barriers and facilitators for the adoption of CPMs in clinical practice.

The patient surveys also included questions about educational levels, which was measured according to the International Standard Classification of Education [48] and health literacy, which was measured with the Set of Brief Screening questions (SBSQ) [49]. The SBSQ assesses perceived difficulties with health information based on three 5-point Likert scale statements ranging from 1–5. An average score of ≤ 3 indicates inadequate health literacy and a score of > 3 adequate health literacy. In the patient survey, the Threatening Medical Situations Inventory (TMSI) was used to assess whether patients handle medically threatening information with either monitoring (attending to the problem) or blunting (avoiding the problem) coping behaviour, since this may affect their views on receiving predictions [50, 51]. In the TMSI, patients are asked how they would handle hypothetical situations. They report on a 5-point Likert scale how likely it would be for them to apply three monitoring and three blunting strategies. Total scores for both the monitoring and blunting strategies are subsequently calculated (ranging from 6–30) [50, 51].

In the nephrologist survey, the Measurement Instrument for Determinants of Innovations (MIDI) was used to identify enablers for the adoption of CPMs in clinical practice [52]. For three domains (the innovation, the user, and the organisation), nephrologists had to pick the two most important determinants that may facilitate the adoption of CPMs in clinical practice. Additional file 3 shows the validated survey instruments used and the study-specific survey questions.

### Pretesting the surveys

Both surveys were tested and amended for face validity by a: 1) communication scientist (CvU), 2) professor of medical decision-making (AS), 3) nephrologist (WB), and 4) cognitive psychologist specialised in communication research (AP). The patient survey was written at the B1 level of the common European framework of reference for languages (CEFRL) to ensure comprehensibility [53]. It was also tested for face validity by five CKD patients recruited by the Dutch Kidney Patients Association.

### Participants, recruitment and informed consent

Patients with CKD were recruited for the interviews by their nephrologists in two Dutch hospitals (St. Antonius hospital and Maasstad hospital) in February 2021. All participants gave informed consent.

For the surveys, CKD patients and nephrologists were recruited from November 2021 until March 2022. Patients were approached via e-mail through the online platform of the Dutch Kidney Patients Association. The nephrologists were approached via e-mail through the online platform of the Dutch Federation for Nephrology. Both surveys were anonymous; no personal identifying information was registered. The patients and nephrologists who agreed to participate were asked to consent with the use of their answers for research and publication purposes when they started the survey. According to the Dutch medical research involving human subjects act, ethical approval was not required for the surveys because participants were not subjected to (medical) procedures or behavioural alternations and the survey was anonymous and limited in its burden (i.e., topics and length).

### Data analysis

All interviews were recorded and transcribed verbatim. The transcripts were coded inductively to identify different themes in the data. One researcher (DH) conducted the primary analysis, which were checked by a second coder (NE). All survey data were analysed with IBM SPSS Statistics (version 28). Descriptive statistics were used to describe the demographic characteristics of the participants. Continuous data are expressed as a mean with standard deviation (SD) or as the median with interquartile range (IQR) when appropriate. Categorical data are presented as valid percent (i.e., percentages when missing data are excluded from the calculations), except for data deriving from multiple answer questions; here absolute frequencies were used. One-way ANOVA or Kruskal–Wallis tests were used (depending on the distribution of the data) to determine whether patients’ mean monitor and blunting scores on the TMSI were associated with patients’ preferences for wanting to know predictions.

## Results

### Semi-structured interviews

Seven CKD patients (four men, three women) with a mean age of 54 years (SD = 15) participated in the interviews. A total of five themes were identified in the data (shown in Table 1). All illustrative quotations can be found in Additional file 4. More than half of the patients (*n* = 5) understood the two predictions visualized in the mock-ups (theme one, understanding predictions about CKD progression). All but one patient indicated they wanted to know both predictions. Three patients preferred the prediction about the time until kidney failure (in years) over the KFRE, and two patients proposed combining them (theme two, preferences for predictions about CKD progression). In theme three ‘how predictions about CKD progression can help patients’, different reasons were mentioned why patients considered these predictions useful. Patients argued that the predictions could: 1) help them with life planning, 2), provide them with more clarity on the stage of their CKD), 3) help them focus on preserving their kidney function for as long as possible, and 4) provide them with comfort or consolation. Potential negative effects of discussing predictions about CKD progression (theme four) included: 1) the predictions could cause increased worrying, and 2) that individual trajectories may vary from the predictions. Lastly, patients indicated how to discuss predictions about CKD progression with patients (theme five). Several patients emphasised that these predictions can be very confrontational and stressed the importance of appropriate guidance and support when the predictions are discussed.

**Table 1:** Identified themes with illustrative quotes from the interviews

Theme	Illustrative quotes
1. Understanding predictions about CKD progression	• *P7 [‘prediction in %*^a^ + *‘prediction in time to*^b^*’]: My initial impression is that this is clear* • *P4: Well, now I see that in 5 years’ time I have a 10% chance of needing kidney replacement therapy and that this isn’t even 3% in two years’ time – what does that add? I don’t understand it very well*
2. Preferences for predictions about CKD progression	• *P6: yeah, it’s about your own health, isn’t it? Why wouldn’t I want to know that? And you indeed realise that, goodness, in nine years’ time I’ll need a donor kidney or kidney dialysis or something of that nature* • *P8: [‘prediction in time to* + *prediction in %’] I feel that it has some relevance. I know, yeah, maybe for some patients that may be something you’d be able to estimate, but… just considering my own case and then to think that I was on the edge and that I’m so much better now. It might not be worth all that much. I mean, yeah, no, that’s a tough one. I don’t know whether I would want to know that, whereas of course other people do want to know that kind of thing*
3. How predictions about CKD progression can help patients	• *P4: [‘prediction in time to’] Of course that would help, because it would help me consider the fact that, well… I guess it’s not that crazy… whether I’d still want to go on another trip or whatever… what would be best: do it now and not in 9 years’ time, because then I’d have to take my dialysis materials with me, or I’d need to have had a kidney transplantation. I mean, yeah, this is… it’s preparing yourself for the fact that you’re going to have to take that step in 9 years’ time* • *P5: [‘prediction in time to’] Yes, yeah, at the times when you’re faced with kidney failure… you do start asking ‘how long do I've got before...?’… especially in relation to how long I’ve got before I need to turn my life upside down. So, erm, yeah, this would definitely help. […] yeah, I would [‘prediction in %* + *prediction in time to’] want to know. That way you’d be able to make or cancel plans. I think that once you’re confronted with kidney failure you really just want to know what the score is*
4. Potential negative effects of discussing predictions about CKD progression	• *P7: Well, what I went through myself is that it was quite a shock when the doctor suddenly told me the [‘prediction in %’]. It’s really… I was in absolute floods of tears, so, yeah, I found the whole thing very, very confronting* • *P8: No, of course, it’ll be different for each patient. That makes sense, in terms of … should I start worrying more or should I start slacking off? Anyway, that is more or less my opinion*
5. How to discuss predictions about CKD progression with patients	• *P9: Well, look, I would want to be told by the nephrologist in any case and if I were able to review that information myself in the future, that would be fine. But if I had no idea whatsoever and then came across this information, I’d be scared out of my mind […] and it’s likely, and this may not even apply to me per se, but if I were to come across this information all at once, I’d want the specialist to tell me that they were keeping an eye on things and recording it in this way* • *P8 Yeah, look, if you’re aware beforehand and know that this information will be adjusted every time… then you might be less shocked. But imagine reading 92%, then I think you would be shocked. I think it’d be better for a doctor to do that. I would only give a patient that result during a consultation – especially if the news is bad*

*CKD* Chronic Kidney Disease

^a^[‘prediction in %’] refers to mock-up of KFRE: % risk to get kidney failure after 2 and 5 years

^b^[‘prediction in time to’] refers to mock-up predicting amount of years until CKD progresses to kidney failure

### Online surveys

In total, 126 out of 407 patients responded to the survey invitation. This amounts to a response rate of 31%. Moreover, 50 out of 438 nephrologists responded to the survey invitation. This amounts to a response rate of 11%. The basic demographics of both the patients and nephrologists are presented in Table 2. The majority of patients (*n* = 113, 90%) had been under nephrology care for at least 5 years. Most patients had undergone kidney transplantation (*n* = 89, 71%) or were not yet on KRT (*n* = 23, 19%). The SBSQ score for health literacy had a median of 4.7 (IQR = 0.7). Most patients (*n* = 100, 79%) were highly educated. Mean scores on the TMSI for monitoring and blunting coping behaviours were comparable, with a mean of 19.4 and 18.6 respectively. At the time of the survey, the nephrologists had been practicing nephrology for a mean of 14.3 years (SD 9.1).

**Table 2:** Demographic characteristics of survey participants

**Patients (*n* = 126)**		
Sex (male), n %	66 (52%)	*Missing 2 (2%)*
Age, median years (IQR)	62 (54–69)	*Missing 3 (2%)*
Education level^a^, n(%)		
Low (levels 0–2)	8 (6%)	*Missing 5 (4%)*
Medium (levels 3–4)	13 (10%)	
High (levels 5–8)	100 (79%)	
SBSQ score, median (IQR)	4.6 (0.7)	
Currently treated in hospital by nephrologist for CKD?		
Yes	122 (97%)	*Missing 2 (2%)*
No	2 (2%)	
How long under nephrology care? n (%)		
< 1 year	3 (2%)	*Missing 4 (3%)*
1–2 years	2 (2%)	
3–5 years	4 (3%)	
> 5 years	113 (90%)	
Current treatment, n (%)		
No KRT	23 (18%)	*Missing 2 (2%)*
Dialysis	10 (8%)	
Peritoneal dialysis	2 (2%)	
Kidney transplantation	89 (71%)	
Conservative care management	0	
Coping strategy threatening information (TMSI)		
Monitor score, mean (SD)	19.4 (4.7)	*Missing 3 (2%)*
Blunter score, mean (SD)	18.6 (3.5)	*Missing 3 (2%)*
**Nephrologists (*n* = 50)**		
Sex (male), n %	29 (58%)	
Age, mean years (SD)	49.2 (8.8)	*Missing 2 (4%)*
Number of years working in current function, mean (SD)	14.3 (9.1)	

All percentages calculated on total population (not valid percentages)

*SD* Standard deviation, *IQR* Interquartile range, *SBSQ* Set of Brief Screening Questions for health literacy, *KRT* Kidney Replacement Therapy, *TMSI* Threatening Medical Situations Inventory

^a^Education levels based on International Standard Classification of Education [48]

### Current use of, and experience with, CPMs

#### Patients

**Fig. 1:**
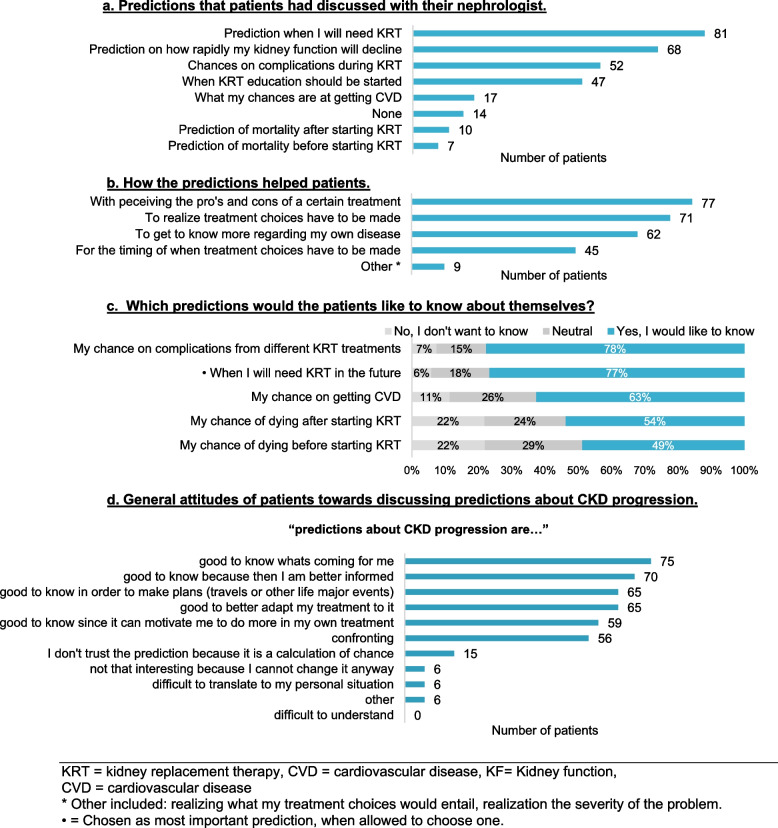
Patients’ experiences with—and preferences in—discussing predictions with their nephrologist. **a** Predictions that patients had discussed with their nephrologist. **b** How the predictions helped patients. **c** Which predictions would the patients like to know about themselves? **d** General attitudes of patients towards discussing predictions about CKD progression. KRT = kidney replacement therapy, CVD = cardiovascular disease, KF = Kidney function, CVD = cardiovascular disease. * Other included: realizing what my treatment choices would entail, realization the severity of the problem. • = Chosen as most important prediction, when allowed to choose one

The majority of patients (*n* = 111, 89%) reported that they had discussed predictions with their nephrologists. The most-commonly discussed predictions were: when they were expected to need KRT (*n* = 81) and how rapidly their kidney function was expected to decline (*n* = 68) illustrated in Fig. 1a. Only two patients indicated that, in retrospect, they would rather not have known these predictions. Patients indicated that discussing these predictions had helped them in the deliberation (pros vs cons) about their KRT options (*n* = 77) and the realization that they had to make a KRT choice (*n* = 71) (illustrated in Fig. 1b).

#### Nephrologists

Just over half of the nephrologists (*n* = 26, 52%) indicated that they used CPMs at the time of the survey. Most nephrologists mentioned using a CPM predicting the risk of cardiovascular disease (CVD) (*n* = 24), followed by a CPM predicting when patients will need KRT (*n* = 8), a CPM predicting the risk of complications associated with different KRT modalities (*n* = 3) and a CPM predicting how blood pressure affects kidney function (*n* = 3). CPM’s predicting mortality before or after starting KRT were mentioned twice. Although a large proportion of nephrologists (*n* = 21, 42%) did not use CPMs or did not know whether they had used them (*n* = 3, 6%), all but two (*n* = 48, 98%) discussed the expected kidney disease trajectory with patients. The majority (*n* = 44, 92%) used graphs of the estimated glomerular filtration rate (eGFR) for this purpose. Nephrologists who did not use CPMs provided different reasons why. The most mentioned reason for not using CPMs was “not knowing any models” (*n* = 11) followed by “not knowing enough about CPMs to use them” (*n* = 6), “not knowing where to find them” (*n* = 4), and “believing currently available CPMS are not reliable enough” (*n* = 4). Less frequently mentioned were “not having enough time to use CPMs during consultations” (*n* = 2), “believing currently available CPMs are impractical and difficult to use” (*n* = 2) and “not seeing the point of using CPMs in providing CKD care” (*n* = 1).

### Preferences for predictions in CKD

#### Patients

Most patients indicated that they wanted to know predictions about: 1) the risk of developing complications associated with the different KRT modalities (*n* = 94, 78%), and 2) when they would need KRT (*n* = 92, 77%) (illustrated in Fig. 1c). When asked to pick the most important prediction, the majority of patients chose “when I will need KRT in the future” (*n* = 42, 61%). Predictions about the risk of dying before or after starting KRT were most frequently chosen as something patients did not want to know (*n* = 27, 22%, and *n* = 26, 22%, respectively). Patients who wanted to know predictions had a significantly higher mean monitoring score compared to those who were neutral, or those who did not want to know these predictions. This was true for patients who desired knowing predictions concerning: 1) the risk of developing CVD (F (2,12) = [10.88], *p* =  < 0.001), 2) when patients would need KRT (F (2,12) = [6.71], p = 0.002), and 3) the risk of dying before starting KRT (F (2,12) = [6.73], *p* = 0.002). The post hoc analyses are provided in Additional file 5. The mean monitoring scores of patients who wanted to know predictions about the risk of developing complications associated with the different KRT modalities, and the risk of dying after starting KRT did not significantly differ from mean monitoring scores of patients who were neutral, or who did not want to know these predictions. There were no significant differences between mean blunting scores as a function of patients’ preferences for wanting to know the different predictions in CKD.

Regarding CPMs about CKD progression, 56 patients indicated that they perceived these predictions as confronting. Nevertheless, patients also agreed that such a prediction could help them to: 1) better know what they can expect (n = 75), 2) become better informed about their CKD (*n* = 70), and 3) help with their (life) planning (*n* = 65) (see Fig. 1d). When patients were shown the mock-up of the prediction from the KFRE, most patients considered it understandable (*n* = 100, 80%). Likewise, most patients (*n* = 105, 84%) understood the mock-up of the prediction in time to kidney failure (in years). The majority of patients wanted to know the prediction from the KFRE (*n* = 89, 72%), 20 (16%) were neutral, and 14 (11%) did not want to know. Similarly, the majority of patients (*n* = 96, 77%) wanted to know the prediction of time to kidney failure (in years), 10 (8%) were neutral, and 18 (15%) did not want to know. Fifty-four patients (45%) preferred the time to kidney failure (in years) prediction compared to 43 (36%) patients preferring the prediction from the KFRE; 24 patients (20%) were neutral. For both predictions, patients indicated that these could help them to: 1) better plan when they have to make a KRT decision, and 2) realize that a KRT decision needs to be made.

#### Nephrologists

**Fig. 2:**
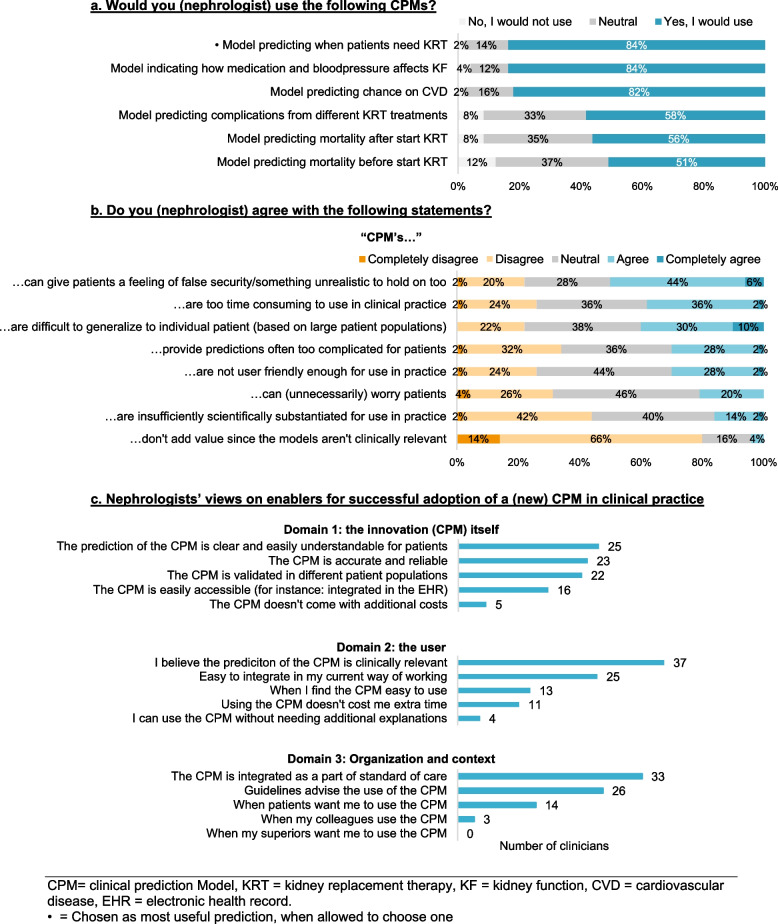
Nephrologists’ preferences and views regarding CPMs. **a** Would you (nephrologist) use the following CPMs? **b** Do you (nephrologist) agree with the following statements? **c** Nephrologists’ views on enablers for successful adoption of a (new) CPM in clinical practice. CPM = clinical prediction Model, KRT = kidney replacement therapy, KF = kidney function, CVD = cardiovascular disease, EHR = electronic health record. • = Chosen as most useful prediction, when allowed to choose one

The nephrologists indicated that they would most likely use a CPM to predict: 1) when CKD patients will need KRT, 2) how medication and blood pressure will affect a patient’s CKD trajectory, and 3) the risk of CVD in patients (illustrated in Fig. 2a). Twenty-three nephrologists (47%) picked a model predicting “when CKD patients will need KRT” as the most useful one. When the nephrologists were asked for what purpose they would want to develop a new CPM, 23 nephrologists (46%) chose “to better inform patients on the expected kidney function trajectory”. Other purposes for developing a new CPM included: “better being able to estimate the effects of treatment on slowing down kidney function deterioration” (*n* = 15, 30%), “better being able to estimate when patients should start KRT education” (*n* = 6, 12%), “better being able to estimate whether or not patients should start a certain kind of KRT” (*n* = 4, 8%) and “better being able to estimate what the expected effects of a certain kind of KRT will be” (*n* = 2, 4%). When they were asked whether they had already used the KFRE in the past, the majority (*n* = 46, 92%) had not; mostly (*n* = 38, 83%) because it was unknown to them. When they were asked whether they would use a CPM to predict the time to kidney failure in years (if available), more than half (*n* = 28, 56%) indicated that they would. The prediction of time to kidney failure (in years) was preferred over the prediction from the KFRE by 31 nephrologists (62%). Four nephrologists explained that they expected patients would better understand a ‘time to’-prediction compared to a ‘risk of’-prediction.

### Barriers and facilitators for the adoption of CPMs in clinical practice

#### Patients

Sixty patients (49%) were neutral on the statement: “nephrologists should use CPMs during their consultations with patients”, 52 (41%) agreed, and 11 (9%) disagreed. Fifty-six patients (46%) wanted nephrologists to explain predictions during consultations, while 45 patients (37%) wanted to view predictions before their consultations so that they could discuss these with their nephrologist. Seventeen patients (14%) wanted to view predictions at any time, regardless of professional guidance.

#### Nephrologists

When the nephrologists were presented with statements arguing against the use of CPMs, the majority agreed that CPMs: 1) can give patients false expectations or a false sense of security (*n* = 22, 50%), 2) don’t say anything about individual patients (*n* = 20, 40%), and 3) are too time-consuming to use (*n* = 18, 38%) (see Fig. 2b). Most nephrologists agreed (*n* = 26, 52%) or completely agreed (*n* = 11, 22%) that CPMs should only be used under professional guidance during consultations, rather than being available for patients at home. The nephrologists were asked to choose two factors from each of the domains of the MIDI (innovation, user, organisation) that they deemed most important in enabling successful use of a (new) prediction model (see Fig. 2c). For domain one (the innovation), the majority of nephrologists (*n* = 25) considered the determinant “The prediction is clear and easily understandable for patients” as the most important determinant for successful adoption in clinical practice. For the second domain (the user), the majority (*n* = 37) considered the determinant “If I believe the prediction from the CPM is clinically relevant” as the most important determinant. For the last domain (the organisation), most (*n* = 33) considered the determinant “The CPM is integrated as a part of standard of care” as the most important determinant for adoption.

All but two nephrologists (*n* = 48, 96%) agreed that they would want to know the performance metrics of CPMs, such as confidence intervals, before they would consider using them. Twenty-three (46%) indicated that they would always discuss these performance metrics with their patients compared to 17 (34%) who would only discuss it with their patients if they believed the patients could understand these metrics and 9 (18%) who would refrain from discussing these metrics because they believed it would be too complicated for patients to understand. About two-thirds of the nephrologists (*n* = 30, 60%) indicated that they would always discuss the uncertainty of an estimated prognosis with their patients, regardless of whether they would use a CPM to make these estimations. Eighteen nephrologists (36%) reported that they would discuss it “in most cases”, one nephrologist (2%) would discuss it “sometimes” and one (2%) would “never” discuss it with patients.

## Discussion

We conducted a national survey study to explore the current use of CPMs in Dutch CKD practice and to identify patients’ and nephrologists’ needs and preferences regarding the use of CPMs, as well as barriers and facilitators for the adoption of CPMs in clinical practice. Even though previous studies suggest that CPMs are used to a limited extent in clinical practice [43, 44], more than half of the nephrologists who participated in our survey reported using CPMs. Likewise, the majority of patients reported that they had discussed predictions with their nephrologist in the past; mostly predictions about their risk of progression to kidney failure. On the contrary, nephrologists reported discussing a CPM for the risk of CVD in patients most frequently. This discrepancy could be explained by the fact that almost all nephrologists reported discussing expected kidney disease trajectories with their patients, and that most of them used graphs of their patients’ eGFR (not a CPM) for this purpose. Patients who participated in this study may have misinterpreted these extrapolations as predictions made with CPMs. For patients, knowing the details of the origin of the prediction might not matter much. However, nephrologists should be aware of this discrepancy when they discuss expected kidney disease trajectories with their patients, since both nephrologists and patients tend to overestimate the risk of progression to ESKD [54].

The majority of both patients and nephrologists advocated for the use of CPMs in CKD practice. These findings are consistent with previous studies [4–6]. Even though a large proportion of patients considered predictions confrontational (particularly predictions about CKD progression), almost none of them regretted discussing predictions with their nephrologists in the past. Reasons for nephrologists why they did not currently use CPMs were most often related to their limited knowledge about, or unfamiliarity with, existing models. Barriers relating to intrinsic motivation, user friendliness or reliability, as often mentioned in the literature [43, 44], were infrequently reported. Perhaps these barriers are overvalued when implementation initiatives are formulated; hindering the widespread adoption of CPMs in CKD practice. Instead, we should focus more on the facilitators for the adoption of CPMs in clinical practice. In this study, facilitators for the adoption of CPMs related to presenting CPMs in a clear and understandable way, incorporating them as a part of standard care, and the CPMs being clinically relevant. Even though previous studies suggest that nephrologists and patients prioritise different treatment outcomes [45], both patients and nephrologists considered CPMs predicting CKD progression as the most relevant prediction, preferably predicting the time to KRT (in years) instead of a 2- and 5-year risk (in %). Patients indicated that this prediction could help them better plan when they have to make a KRT decision and realize that a KRT decision has to be made. The latter is an important enabler for patient empowerment in starting a shared decision-making process [55].

When we explored patients’ normative beliefs about whether or not nephrologists should use CPMs during consultations, most were neutral or agreed that they should. However, it should be noted that there was a small proportion of patients who did not want to know any predictions when we explored their preferences for both CPMs in general, and CPMs related to CKD progression. This is especially relevant considering that the participating patients are potentially taking on a more active role in treatment decision-making compared to the general patient population (since they were highly educated, had high health literacy and were recruited from the Dutch Kidney Patients Association). The actual number of patients that do not want to know these predictions could potentially be higher in clinical practice. Although we did identify that higher monitor scores might be associated with wanting to know certain predictions, we did not find higher monitor scores in our study population when compared to their individual blunting scores, or to scores from other studies [50, 56]. Similar to others who studied patient preferences for receiving prognostic information [57], we propose that nephrologists simply ask, and provide patients with the opportunity to make their own decisions about whether or not they want predictive information to be shared with them.

In addition to the highly educated patient population, the majority of the patients included in this study were patients who had received a kidney transplant and were under treatment for more than 5 years with their nephrologist. This affects generalization of the results towards the whole CKD population. Hypothetically, patients earlier in their disease phase might have different information needs regarding the use of CPMs. Additionally, participating patients might have discussed the predictions regarding CKD progression a longer time ago, increasing changes on recall bias. For the clinician’s survey, issues with generalization should also be noted; these survey results may not be indicative for all Dutch nephrologists. Since the response rate to the survey was low, we cannot exclude non-response bias. Nephrologists who were willing to fill in the survey may hold more positive attitudes towards CPMs than nephrologists who didn’t.

We are among the first to provide quantitative data on what both patients and nephrologists prefer regarding the use and purpose of CPMs, and what predictions they prioritise. Moreover, we collected information on important determinants for the successful adoption of CPMs in clinical practice, which may be used to guide the implementation of CPMs. In addition, researchers and developers can use our findings for improving existing CPMs or for developing new CPMs. When the latter is considered, our study shows that patients and nephrologists prefer a ‘time to kidney failure’ prediction, rather than a ‘risk of progression to kidney failure’ prediction. This study focused on currently available CPMs in CKD. Future research may explore newly developed CPMs, such as CPMs predicting patient reported outcomes.

## Conclusion

In this study, both nephrologists and the majority of patients want to discuss CPMs in Dutch CKD practice, especially those that predict CKD progression. Validated and freely available CPMs, that largely meet the needs and preferences expressed by patients and nephrologists in this study, already exist (e.g. the KFRE). However, these CPMs appear to be underused due to lack of knowledge regarding where to find them and how to use them meaningfully. We should focus on improving the accessibility of these CPMs and provide guidance on how to communicate the predictions effectively. Additionally, whether or not patients want to hear particular predictions varies among individual patients, and their preferences should therefore be explored during consultations.

## Supplementary Information

**Additional file 1: Figure S1.** Mock-ups of two predictions of models predicting CKD progression (translated from Dutch).

**Additional file 2: Figure S2.** Infographic explaining a clinical prediction model (in Dutch).

**Additional file 3: Table S1.** Content of the online surveys for patients and nephrologists.

**Additional file 4: Table S2.** Identified themes and illustrative quotes from patient interviews.

**Additional file 5: Box S1.** Post-hoc analysis of coping strategies in relation to preferences regarding CPMs.

## Data Availability

The datasets used and/or analysed during the current study are available from the corresponding author on reasonable request.
