# Supplementary material for: Predicting outcomes in chronic kidney disease: needs and preferences of patients and nephrologists
Source: BMC Nephrol. 2023 Mar 22;24:66. doi: 10.1186/s12882-023-03115-3 (PMC10035227; doi:10.1186/s12882-023-03115-3)
Supplement: Supplementary file 1 — Additional file 1: Figure S1. Mock-ups of two predictions of models predicting CKD progression (translated from Dutch). [file 12882_2023_3115_MOESM1_ESM.docx]

**Supplement Figure S1: Mock-ups of two predictions of models predicting CKD progression** *(translated from Dutch)*

Mock-up 1:


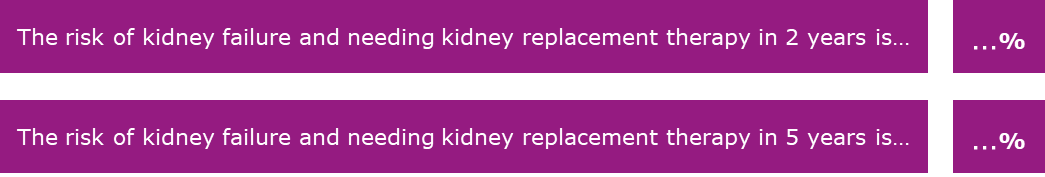


Mock-up 2:

You might need kidney replacement therapy because of kidney failure in … **years**
