# Supplementary figures and images for: Predicting outcomes in chronic kidney disease: needs and preferences of patients and nephrologists
Source: BMC Nephrol. 2023 Mar 22;24:66. doi: 10.1186/s12882-023-03115-3 (PMC10035227; doi:10.1186/s12882-023-03115-3)

**Supplement Figure S2: Infographic explaining a clinical prediction model** *(in Dutch)*


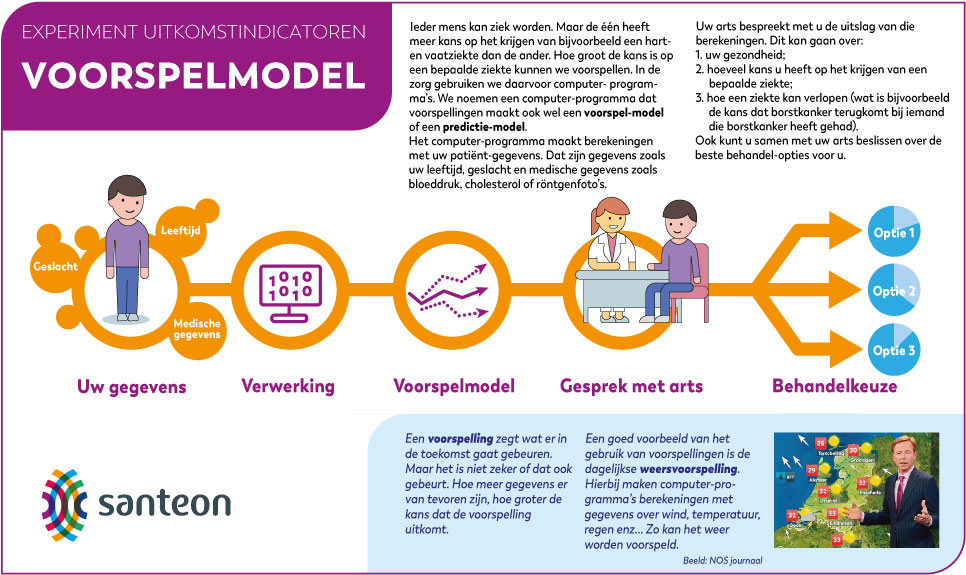

Supplement: Supplementary file 2 — Additional file 2: Figure S2. Infographic explaining a clinical prediction model (in Dutch). [file 12882_2023_3115_MOESM2_ESM.docx]
