# Supplementary material for: Predicting outcomes in chronic kidney disease: needs and preferences of patients and nephrologists
Source: BMC Nephrol. 2023 Mar 22;24:66. doi: 10.1186/s12882-023-03115-3 (PMC10035227; doi:10.1186/s12882-023-03115-3)
Supplement: Supplementary file 3 — Additional file 3: Table S1. Content of the online surveys for patients and nephrologists. [file 12882_2023_3115_MOESM3_ESM.docx]

**Supplement Table S1:** Content of the online surveys for patients and nephrologists.

| **Topics** | **Patient survey** | **Nephrologist survey** |
| --- | --- | --- |
| **Introduction** | Definition of a ‘prediction model’ including infographic | |
| **Demographics** | - Age - Sex - Education level^1^ - Health literacy (SBSQ)^2^ - Estimated remaining KF at time of survey - Coping behaviour (TMSI)^3^ | - Age - Sex - Professional experience in current function (in years) |
| **Current use of CPMs in CKD practice** | - Did your nephrologist discuss predictions with you? If so: which one(s)? - Retrospectively; did you want to know these prediction(s)? | - Do you currently use CPMs? If yes: which one(s)? - If not: reasons not to use CPMs? - Do you discuss predictions without using CPMs? If yes: how do you discuss these expectations? |
| **Preferences for predictions in CKD** | - Which prediction(s) (drawn from the literature) do you want to know (and why)? - What do you consider the most important prediction (and why)? - How can CPMs be helpful to you? | - Which CPMs (drawn from literature) would you want to use in the future? - What do you consider the most important prediction (and why)? - For what purpose would you develop a new CPM if anything is possible? |
| **Preferences for predictions about CKD progression** | Mock-ups of 2 CPMs:  1) the KFRE; a two- and five-year risk prediction of progression to kidney failure (in %)  2) prediction of the time to progression in kidney failure (in years) | |
|  | - Do you understand both predictions? - Would you want to know this information about yourself? - Which prediction do you prefer? | - Have you used the KFRE? - Would you use these predictions? - Which CPM do you prefer? |
| **Barriers and facilitators for the adoption of CPMs in clinical practice** | - Testing general attitudes (drawn from interviews) when hearing prediction models - Do you think nephrologists should use CPMs during consultations? - How/when should predictions be communicated? | - Do you agree with statements (drawn from the literature) arguing against the use of CPMS in clinical practice? - Which determinants of the MIDI^4^ are most important for the successful adoption of CPMs in clinical practice? - How/when should predictions be communicated? |

CPMs = clinical prediction models, CKD = chronic kidney disease, KRT = Kidney replacement therapy

1 = The International Standard Classification of Education framework was used to present patients’ educational levels [48]
2 = Set of Brief Screening Questions [49]

3 = Threatening Medical Situations [50,51]

4 = Measurement Instrument for Determinants of Innovations [52]
