# Supplementary material for: Predicting outcomes in chronic kidney disease: needs and preferences of patients and nephrologists
Source: BMC Nephrol. 2023 Mar 22;24:66. doi: 10.1186/s12882-023-03115-3 (PMC10035227; doi:10.1186/s12882-023-03115-3)
Supplement: Supplementary file 4 — Additional file 4: Table S2. Identified themes and illustrative quotes from patient interviews. [file 12882_2023_3115_MOESM4_ESM.docx]

**Supplement Table S2: Identified themes and illustrative quotes from patient interviews**

| **Themes** | **Summarised key points** | **Quotes**  **[****prediction in %] refers to KFRE: % risk to get kidney failure after 2 and 5 years.**  **[prediction in time to] refers to predicting amount of years until CKD progresses to kidney failure.** |
| --- | --- | --- |
| 1. **Understanding predictions about CKD progression** | No difficulties in understanding outcomes of the CPMs | P1: Let me just read… what I understand from it is that it shows what things will look like in 2 years or in 5 years.  P5 Erm, yeah, I think this is especially for those who haven’t had a transplant before or those where kidney replacement therapy hasn’t been initiated before… because that’s what this is about, isn’t it – about when that time comes?  P6: yes, yes. It’s clear what it says.  P7 [‘prediction in % + ‘prediction in time to’]: My initial impression is that this is clear.  P4 [‘prediction in time to’]: The latter refers to in 9 years’ time. Yes, well, this patient has been aware of the fact that he has been suffering from kidney disease since 2016, so it’s a good thing to be able to give someone a timeline. |
|  | Difficulty in understanding outcomes of the CPMs | P4: [‘prediction in %’] Okay, the likelihood of kidney failure and needing kidney replacement therapy in 2 years’ time is 2.63% and the likelihood of kidney failure and needing kidney replacement therapy in 5 years’ time. No, actually, I feel this is a vague figure.  P4: Well, now I see that in 5 years’ time I have a 10% chance of needing kidney replacement therapy and that this isn’t even 3% in two years’ time – what does that add? I don’t understand it very well. |
| 1. **Preferences for predictions about CKD progression** | Willingness to know predictions about CKD progression | P4: It is what it is and you do understand that it is a prediction based on the things that you have provided and the data the doctor gets from the tests. And yeah, it’s just useful to know which way you’re going.    P5: [‘prediction in %’] If you haven’t experienced that before, it can be very nerve-racking. However, I can imagine that you would, for instance, want to know how you’re doing and what your chances are.  P6: yeah, it’s about your own health, isn’t it? Why wouldn’t I want to know that? And you indeed realise that, goodness, in nine years’ time I’ll need a donor kidney or kidney dialysis or something of that nature.  P7: [‘prediction in %’] this is relevant to everyone, the percentages you are likely to need kidney replacement therapy in two years’ or five years’ time. Yeah, that’s just very important.  P9: [‘prediction in %’I would definitely want to know, because if, at some point, I was told that, but it was already at 3.62 three years ago – for example – well, then I would have liked to know. Definitely! |
|  | Unwillingness to know predictions about CKD progression | P8: [‘prediction in time to + prediction in %’] I feel that it has some relevance. I know, yeah, maybe for some patients that may be something you’d be able to estimate, but… just considering my own case and then to think that I was on the edge and that I’m so much better now. It might not be worth all that much. I mean, yeah, no, that’s a tough one. I don’t know whether I would want to know that, whereas of course other people do want to know that kind of thing. |
|  | No preferences between a ‘risk of’ prediction or a ‘time to’ prediction format | P1: [‘prediction in time to + prediction in %’] It’s basically the same screen as before, only it says 9 years, so it’s… yeah… in more detail… the whole thing deals with multiple years. Other than that, it’s actually exactly the same – so it’s not a preference.  P9: [‘prediction in time to + prediction in %’] Erm, of course that’s very different, because for one person it’ll be 2 or 5 years and then a percentage; and in this case we’re talking about 9 years. So those nine years, to my mind, come across as more positive than 2 or 5 years. On the other hand, I’d prefer clear information and you shouldn’t sugarcoat things to be better than they actually are. If it is actually nine years then… fine. But maybe that percentage is equally fine. I’d be okay with either of them, provided I know that I can expect something in future. |
|  | Preference for the ‘time to’ prediction | P8: Hmm, well, maybe a prediction saying ‘’ in the amount of years [‘prediction in time to’] may be a bit clearer than in percentages [‘prediction in %’].  P4: Surely, it would be more relevant to know when kidney replacement therapy is indeed necessary [‘prediction in time to’]. Look at the possibilities of kidney replacement therapy in 9 years’ time, I mean, yeah, that’s exactly the questions you’re asking.  P5: Ah, yeah, saying ‘x amount of years’, might be much better, as it’s just 1 number. And, erm, look, percentages are quite abstract – it tells you your chances, erm, in terms of that you might need it in 9 years’ time. |
|  | Preference for a combination of a ‘risk of’ and a ‘time to’ prediction | P7: I would really like it if, say, this could be combined, as it were, meaning that you have the ‘in 9 years’ time’ plus the percentages outlined alongside it.  P6: maybe you could put this bar [‘prediction in time to’] there and [‘prediction in %’] underneath. That would give you an overall picture. That would give you the percentages and the number of years. That might be clearer for people? |
| **3. How predictions about CKD progression can help patients** | Predictions about CKD progression can help patients with their life planning | P4: [‘prediction in time to’] Of course that would help, because it would help me consider the fact that, well… I guess it’s not that crazy… whether I’d still want to go on another trip or whatever… what would be best: do it now and not in 9 years’ time, because then I’d have to take my dialysis materials with me, or I’d need have to have had a kidney transplantation. I mean, yeah, this is… it’s preparing yourself for the fact that you’re going to have to take that step in 9 years’ time.  P5: [‘prediction in time to’] Yes, yeah, at the times when you’re faced with kidney failure… you do start asking ‘how long have I got before?’… especially in relation to how long I’ve got before I need to turn my life upside down. So, erm, yeah, this would definitely help. […] yeah, I would [‘prediction in % + prediction in time to’] want to know. That way you’d be able to make or cancel plans. I think that once you’re confronted with kidney failure you really just want to know what the score is.  P7: [‘prediction in %’] Well, it might help me with regard to the expectations I have for the future and equally what I’ve been discussing with my doctor recently… about having children. I mean, I am very young, but the expectation is that between now and 6 years I should be having a kidney transplantation. And yeah, imagine you want to start planning starting a family, then, in my case, it would be highly relevant… in terms of, well, I should have my kidney transplant first… and if I were to know that it would roughly be… in this case it would be in about 5 years… 9.% or 80%… then I’d have rough idea of where I stand and, yeah, that’s just something that’s good to know. |
|  | Predictions about CKD progression can provide patients more clarity on their disease stage | P9: well, I don’t know whether it’d be helpful, but it is clear. I don’t know what would be beneficial to me or how it would help me. The only thing that is clear is what stage I’m at.  P7: yeah, imagine discussing this with your parents… my parents also know quite a lot about kidney failure and such, so they might know a bit more about this… and it would make more sense to them in terms of a percentage. But imagine I were talking about it to my friends and I guess it would make less sense to them… they’d find it more logical to speak in terms of 5 to 6 years – that would give them a clearer idea. |
|  | Predictions about CKD progression can provide patients with comfort or consolation. | P5: So, the chance of kidney failure and needing kidney replacement therapy in two years’ time is 2.63%… that’s very reassuring to read, so yeah… I think that’s very useful. |
|  | Predictions about CKD progression can help patients focus on preserving their kidney function for as long as possible | Interviewer: And could that information [‘prediction in % + prediction in time to’] help you?  P6: yes, you could… the only thing you could do is discuss things with your doctor… in terms of what you could do even better  P6: [‘prediction in % + prediction in time to’] yeah, it’s about your own health, isn’t it? Why wouldn’t I want to know that? And you indeed realise that, goodness, in nine years’ time I’ll need a donor kidney or kidney dialysis or something of that nature. Erm, yes. What can I do in the meantime to stretch out that period somewhat? |
| 1. **Potential negative effects of discussing predictions about CKD progression** | Predictions about CKD progression can be very confrontational | P9: I’m also very curious to see how things are in 5 years’ time. What percentage I’d have. […] It makes me a little anxious thinking about it. I’ll  say quite honestly, I’ve never really thought about it that way before. I’m finding this a little difficult [tearing up]   P9: yeah – that was a bit of a shock. If that was for me, I’d really have had a scare. I’d think I probably should go back to the Netherlands.  P7: Well, what I went through myself is that it was quite a shock when the doctor suddenly told me the [‘prediction in %’]. It’s really… I was in absolute floods of tears, so, yeah, I found the whole thing very, very confronting.  P9: It just surprises me… woah! We’ve never talked about this before. I mean, yeah, if it’s below 20, then we would have more serious conversations. But I think it’s at 35, so I’m still way over the halfway mark. So yeah, I’m trying to live as healthy a life as possible and am not giving it any further thought – but when I saw the 2- and 5-year points, I just thought: woah! That’s pretty intense. And those aren’t even my own numbers. |
|  | Predictions about CKD progressions can cause increase worrying and/or can be unmotivating | P8: No, of course, it’ll be different for each patient. That makes sense, in terms of … should I start worrying more or should I start slacking off? Anyway, that is more or less my opinion. |
| **5. How to discuss predictions about CKD progression with patients** | Predictions about CKD progression should be discussed with a nephrologists (especially the first time) | P7: Well, what I went through myself is that it was quite a shock when the doctor suddenly told me the [‘prediction in %’]. It’s really… I was in absolute floods of tears, so, yeah, I found the whole thing very, very confronting. I would find it even more distressing if I were to see that on the site for the first time.  P8 Yeah, look, if you’re aware beforehand and know that this information will be adjusted every time… then you might be less shocked. But imagine reading 92%, then I think you would be shocked. I think it’d be better for a doctor to do that. I would only give a patient that result during a consultation – especially if the news is bad.  P9: Well, look, I would want to be told by the nephrologist in any case and if I were to be able to review that information myself in the future, that would be fine. But if I had no idea whatsoever and then came across this information, I’d be scared out of my mind […] and it’s likely, and this may not even apply to me per se, but if I were to come across this information all at once, I’d want the specialist to tell me that they were keeping an eye on things and recording it in this way.  P9: So, in the consultation with my nephrologist, he might say, well, this is the picture now, considering your situation, and this and that and he says it’ll be 25% in 5 years’ time. And then if I were to check the next time and see 21% come up and then think to myself ‘oh, it’s starting to fall’. But if it happened to be 27% the next time, which is equally possible, I’d think ‘well, hey! I suspect that it may very well still be 25% in 5 years’ time, but as long as I hear the nuts and bolts of it from my specialist first and am able to take a look at the information myself, then I wouldn’t mind at all.  P5: Yeah – I would like to be able to review things at home before seeing the nephrologist. You’ll most likely only be given access once you’ve already been to see the nephrologist and already have symptoms and there have already been problems – erm, yeah, from that moment onwards I just want to have everything be clear to me. |
|  | When discussing these predictions, it has to be clear that it is relates to an estimate | P7: [‘prediction in time to’] Don’t make the test definitive, meaning that in this case kidney replacement therapy would be necessary in 9 years’ time, but that it is actually an estimate… that has to be made very clear. |
