# Supplementary material for: Predicting outcomes in chronic kidney disease: needs and preferences of patients and nephrologists
Source: BMC Nephrol. 2023 Mar 22;24:66. doi: 10.1186/s12882-023-03115-3 (PMC10035227; doi:10.1186/s12882-023-03115-3)
Supplement: Supplementary file 5 — Additional file 5: Box S1. Post-hoc analysis of coping strategies in relation to preferences regarding CPMs. [file 12882_2023_3115_MOESM5_ESM.docx]

**Supplement Box 1 S1:** Post-hoc analysis of coping strategies in relation to preferences regarding CPMs

Post hoc comparison using the Tukey HSD test indicated that mean monitoring score for *wanting to know* the **prediction regarding the chance of getting CVD** was significantly different than *not wanting to know* (mean difference 4.42, 95% BI (1.40-7.45) and being *neutral* (mean difference 3.50, 95%BI 1.31-5.70).

Post hoc comparison using the Tukey HSD test indicated that mean monitoring score for *wanting to know* the **prediction regarding when patients might need KRT** was significantly different than being *neutral* (mean difference 4.07, 95%BI 1.41-6.73).

Post hoc comparison using the Tukey HSD test indicated that mean monitoring score for *wanting to know* the **prediction regarding the chance of mortality before KRT** was significantly different than *not wanting to know* (mean difference 3.24, 95%BI 0.75-5.74) and being *neutral* (mean difference 2.81, 95%BI 0.52-5.09)
